# Trash or Treasure: extracellular microRNAs and cell-to-cell communication

**DOI:** 10.3389/fgene.2013.00173

**Published:** 2013-09-05

**Authors:** Nobuyoshi Kosaka, Yusuke Yoshioka, Keitaro Hagiwara, Naoomi Tominaga, Takeshi Katsuda, Takahiro Ochiya

**Affiliations:** Division of Molecular and Cellular Medicine, National Cancer Center Research InstituteTokyo, Japan

**Keywords:** circulating microRNA, exosomes, extracellular vesicles, extracellular microRNA, secretory microRNA, cell-to-cell communication

## Abstract

Circulating RNAs in human body fluids are promising candidates for diagnostic purposes. However, the biological significance of circulating RNAs remains elusive. Recently, small non-coding RNAs, microRNAs (miRNAs), were isolated from multiple human body fluids, and these “circulating miRNAs” have been implicated as novel disease biomarkers. Concurrently, miRNAs were also identified in the extracellular space associated with extracellular vesicles (EVs), which are small membrane vesicles secreted from various types of cells. The function of these secreted miRNAs has been revealed in several papers. Circulating miRNAs have been experimentally found to be associated with EVs; however, other types of extracellular miRNAs were also described. This review discusses studies related to extracellular miRNAs, including circulating miRNAs and secreted miRNAs, to highlight the importance of studying not only secreted miRNAs, but also circulating miRNAs to determine the contribution of extracellular miRNAs especially in cancer development.

## INTRODUCTION

Circulating RNAs have been isolated from human body fluids (Kamm and Smith, 1972; Fleischhacker and Schmidt, 2007). Javillier and Fabrykant (1931) reported the first discovery of circulating nucleic acids in 1931, before Watson and Crick (1953) reported the structure of DNA as a double helix. Furthermore, Mandel and Metais (1947) permitted ribonucleic acid and deoxyribonucleic acid to be separately measured. Since then, many researchers have attempted to use circulating RNA as disease biomarkers; however, the origins and meanings of circulating RNA are poorly understood.

MicroRNAs (miRNAs) are small non-coding RNAs that regulate multiple phenomena, including development, organogenesis, and homeostasis (Ebert and Sharp, 2012). The mis-expression of miRNAs results in the onset of diseases, such as immune disease, cardiovascular disease, neurological disease, and cancer (Mendell and Olson, 2012). In 2007, the Lötvall group demonstrated that miRNAs were contained inside exosomes (Valadi et al., 2007), which are small membranous vesicles derived from the endosome (Raposo and Stoorvogel, 2013). Since the discovery of miRNAs in exosomes, several reports confirmed the existence of miRNAs in apoptotic bodies (Zernecke et al., 2009), high-/low-density lipoprotein (HDL/LDL; Vickers et al., 2011), and RNA-binding proteins (Arroyo et al., 2011; Turchinovich et al., 2011). Other studies have shown the existence of circulating miRNAs in human serum, including the serum from pregnant women (Chim et al., 2008) and cancer patients (Lawrie et al., 2008). Researchers have identified placental-specific miRNAs in the serum from pregnant women, which clearly disappeared after childbirth, indicating that circulating miRNAs reflect the status of the individual (Chim et al., 2008). Similarly, cancer-associated miRNAs were higher in the serum from cancer patients than in the serum from healthy individuals, indicating that circulating miRNAs can be used as biomarkers to monitor the existence of cancer cells in patients (Lawrie et al., 2008). These reports also demonstrated the stability of circulating miRNAs in the blood, despite the presence of large amounts of RNase (Reddi and Holland, 1976). Since the discovery of miRNAs in blood, many researchers have confirmed the existence of miRNA in a variety of other human body fluids, such as serum, plasma, saliva, breast milk, urine, and cerebrospinal fluid, among others (Kosaka et al., 2010a).

In this review, we chose miRNAs that were reported to have functions in cell–cell communication and also reported to be a potential biomarker, and we attempted to link the findings concerning secreted miRNAs used in cell–cell communication tools and circulating miRNAs used as biomarkers. This discussion may increase broad interests and improve the current understanding of the importance of extracellular miRNAs in cell–cell communication. We would like to discuss about the vesicles, such as exosomes, microvesicles, and apoptotic bodies (Bobrie et al., 2011; Raposo and Stoorvogel, 2013). The mean size of exosomes, 40–100 nm in diameter, corresponds to that of the internal vesicles of multivesicular bodies from which they originate. Exosomes contain enriched amounts of some specific markers, especially those of endosomal origin including CD63, CD81, CD9, major histocompatibility complex class II, and so on. On the other hand, the size of microvesicles varies between 50 nm and 1 μm in diameter and the microvesicles are generated by budding at the plasma membrane toward the outside of the cell. However, the term of microvesicles has also been used for exosome-like vesicles and clear distinction of exosome and microvesicles has not been established; therefore, we will use “extracellular vesicle (EV)” in this review, according to the definition of the International Society for Extracellular Vesicles, when describing studies using ultracentrifugation to isolate EVs.

## miRNAs IN EXTRACELLULAR VESICLES OR NON-VESICLE ASSOCIATED miRNAs

It has been shown that EVs, such as exosomes, microvesicles, and apoptotic bodies, contain miRNAs with functions that have been previously reported (Valadi et al., 2007; Zernecke et al., 2009). The existence of non-vesicle associated miRNAs has also been reported. These miRNAs bind to HDL/LDL (Vickers et al., 2011) or RNA-binding proteins, such as Argonaute 2 (Ago2) (Arroyo et al., 2011; Turchinovich et al., 2011) and Ago1 (Turchinovich and Burwinkel, 2012). Interestingly, Arroyo et al. (2011) reported that circulating miRNAs in plasma are predominantly coupled with Ago2. The liver-specific miRNA, miR-122 has been detected only in protein-associated fractions, suggesting that hepatocytes might release miR-122 through a protein carrier pathway. In addition, Turchinovich and Burwinkel (2012) showed that not only Ago2 but also Ago1-bound miRNAs has been identified in human blood plasma. Intriguingly, they also found that some miRNAs in the plasma did not derive from blood cells under normal conditions. Although the abundance of miRNAs associated with RNA-binding proteins has been recognized, the functions of these miRNAs in cell–cell communications have not been clarified.

## miR-210

miR-210 is a hypoxia-inducible miRNA that is activated by the master regulator of hypoxic stress, hypoxia-inducible factor (HIF)-1alpha in a variety of cell types (Chan et al., 2012). This miRNA has been implicated in erythropoiesis (Kosaka et al., 2008), iron homeostasis (Yoshioka et al., 2012), angiogenesis (Fasanaro et al., 2008), and cancer (Huang et al., 2009), which are also conditions associated with hypoxic stress. This miRNA has also been implicated in the regulation of DNA repair pathways (Crosby et al., 2009). The function of miR-210 has been investigated, although its exact contribution to the cancer microenvironment has not been determined.

Recently, we observed that EVs isolated from metastatic breast cancer cells promote metastasis via the induction of angiogenesis in the tumor (Kosaka et al., 2013). We also showed that EVs contain multiple angiogenic miRNAs, and one of them, miR-210, is responsible for angiogenesis. Indeed, the addition of miR-210-enriched EVs induced the activation of endothelial cells *in vitro* (Kosaka et al., 2013). Moreover, miR-210 expression is known to be inversely correlated with a disease-free and overall survival in breast cancer (Camps et al., 2008). Intriguingly, circulating miR-210 in breast cancer patients has been reported. The expression of circulating miR-210 is significantly higher in plasma from circulating tumor cell (CTC)-positive metastatic breast cancer patients compared with that in plasma from CTC-negative metastatic breast cancer patients and controls (Madhavan et al., 2012). The use of CTC as a prognostic marker in metastatic breast cancer has been well documented (Lianidou and Markou, 2011); however, adequate detection methods are still needed. Thus, circulating miRNAs could be used to predict the status of patients with metastatic breast cancer instead of detecting CTC. Moreover, the indication of CTC is associated with bad prognosis for cancer patients, and circulating miR-210 might contribute to this phenomenon (Madhavan et al., 2012).

Interestingly, circulating miR-210 levels were significantly higher in individuals with residual disease than in those who achieved a pathologically complete response to trastuzumab (Jung et al., 2012), administered at baseline before patients received neoadjuvant chemotherapy, as a part of the standard treatment for patients with human epidermal growth factor receptor 2 (HER-2)-positive breast cancer. Indeed, circulating miR-210 was derived from tumor cells, as reduced levels of circulating miR-210 were observed in the serum of patients after surgery compared with that in serum from patients before surgery. Furthermore, miR-210 expression was also higher in patients whose cancer metastasized to the lymph nodes. These results suggest that circulating miR-210 can be used to predict and perhaps monitor responses to therapies involving the use of trastuzumab. Elevated levels of HIF-1alpha were also associated with HER-2 over-expression in invasive breast cancer (Yamamoto et al., 2008). Moreover, the induction of HER-2 signaling in breast cancer cells increases HIF-1alpha protein and vascular endothelial growth factor (VEGF) mRNA expression (Laughner et al., 2001).

Taken together, these results suggest that miR-210 contributes to cancer development through immediate effects on the cancer cells and the modulation of the cancer cell microenvironment, and when secreted into peripheral blood, circulating miR-210 can be detected to predict the status of cancer cells in the tumor (**Table 1**).

**Table 1:** The miR-210 studies in the cells and in the extracellular space.

Location	Phenotype	Origin of miR-210 expression	Reference
Intracellular	Anti-apoptosis in erythroid cells	Erythroid cells	Kosaka et al. (2008)
Intracellular	Regulate iron homeostasis by targeting ISCU and TfR1	Breast cancer cells	Yoshioka et al. (2012)
Intracellular	Regulate response to hypoxia by suppressing Ephrin-A3	Endothelial cells	Fasanaro et al. (2008)
Intracellular	Regulating the hypoxic response of tumor cells and tumor growth	Renal cancer cells	Huang et al. (2009)
Intracellular	Promote genetic instability via suppression of RAD52	Cervical carcinoma cells and breast cancer cells	Crosby et al. (2009)
Extracellular (endothelial cells)	Promote metastasis via the induction of angiogenesis through EVs delivery	Metastatic breast cancer cells	Kosaka et al. (2013)
Extracellular (blood)	High expression in serum from patients who have trastuzumab-resistance cancer	Drug resistance breast cancer cells	Jung et al. (2012)
Extracellular (blood)	High expression in CTC-positive patient	Breast cancer cells	Huang et al. (2009)

*EVs, extracellular vesicles; ISCU, iron –sulfur cluster scaffold; TfR1, transferrin receptor 1; CTCs, circulating tumor cells; EPO, erythropoietin.*

## EBV miRNAs

Epstein–Barr virus (EBV) encodes miRNAs, which were first reported viral miRNAs in human. A recent study on EBV-infected normal and neoplastic tissues revealed that distinct EBV miRNA expression profiles are produced in various latency programs, and EBV miRNAs play key roles in maintaining EBV persistence through the inhibition of apoptosis and the suppression of the host immune response (Forte and Luftig, 2011).

Previously, Pegtel et al. (2010) observed that functional EBV miRNAs, secreted from EBV-infected cells, are transferred to uninfected recipient cells. These authors showed the miRNA-mediated repression of confirmed EBV target genes, including *CXCL11*. Importantly, in a co-culturing system, containing EBV-transformed lymphoblastic B cells (donor cells) and primary immature monocyte-derived dendritic cells (recipient cells), approximately 2 × 10^3^ copies of EBV-miRNA BART1-5p were detected in a subset of the recipient cells after 24 h, and this level increased fourfold (nearly 8 × 10^3^ copies) after an additional 24 h of co-culture. Moreover, these authors confirmed that the expression of *CXCL11* in recipient cells was down-regulated within 24 h co-culture, suggesting that the transfer of 2 × 10^3^ copies of EBV-miRNA is sufficient to suppress miRNA-target genes in recipient cells. Surprisingly, EBV miRNAs were present in both B cell and non-B cell fractions in peripheral blood mononuclear cells obtained from patients with an increased EBV load, although EBV DNA was restricted to the circulating B cell population. These observations indicated that viral miRNAs are functional in non-infected cells after the transfer of virus miRNAs from infected cells to non-infected cells through EVs. As shown above, this study provided the quantitative information on the level of extracellular miRNAs, which is essential for research on exosomal miRNA-mediated cell–cell communication. Information, such as the level of exosomal miRNAs required to suppress target molecules in recipient cells, might improve the quality of research on exosomal miRNAs in cell-cell communications.

Nasopharyngeal carcinoma (NPC) is a human epithelial malignancy associated with EBV, and EBV miRNAs are abundantly found in NPC tumors (Lo et al., 2012). Interestingly, viral miRNAs are secreted into the extracellular space from NPC cells with secreted EVs (Gourzones et al., 2010). In addition, these miRNAs are not only detected in plasma samples from NPC xenografted nude mice, but also in plasma samples from NPC patients. Moreover, EBV miRNAs were significantly up-regulated in tumor tissues compared with non-tumor biopsies, and the distinct presence of EBV miRNAs in the serum of NPC patients has been positively correlated with the cellular copy numbers of EBV miRNAs (Wong et al., 2012). Taken together, these results indicated that the viral miRNAs secreted from NPC cells, are contained inside EVs, resulting in the high stability for diffusion from the tumor site to the peripheral blood.

Interestingly, non-infected cells harbor miRNAs from viruses, and this fact might be an important aspect to reconsider infectious diseases. In the case of NPC, several studies have shown the contribution of EBV miRNAs to cancer development (Lo et al., 2012), and circulating miRNAs might be useful for the evaluation of patient status (Gourzones et al., 2010; Wong et al., 2012). Considering the delivery of EBV miRNAs through EVs, it is important to characterize the roles of EBV miRNAs in “non-infected cells” during the development of NPC. Moreover, miRNAs have been identified in numerous virus types, such as herpes B virus, human cytomegalovirus, herpes simplex virus, and Kaposi’s sarcoma-associated herpes virus, among others. Thus, it would be important to examine the roles for these viral miRNAs in non-infected cells. This information might broaden the current understanding of infectious diseases caused by virus miRNAs.

## miR-21

miR-21 is a well-characterized miRNA that contributes to the development of cancer (Schetter et al., 2008; Medina et al., 2010), and the target genes for miR-21 have been identified as well-known tumor suppressor genes, such as PTEN (Meng et al., 2007) and PDCD4 (Asangani et al., 2008). Thus, it is natural to examine the expression of circulating miR-21 in the serum of cancer patients for diagnosis. Indeed, several reports have shown the increased expression of circulating miR-21 in the serum of cancer patients, including diffuse large B cell lymphoma (DLBCL; Lawrie et al., 2008), osteosarcoma (Ouyang et al., 2013), colorectal cancer (Kanaan et al., 2012), hepatocellular carcinoma (HCC; Zhou et al., 2011), gastric cancer (Li et al., 2012), head and neck squamous cell carcinoma (Hsu et al., 2012), esophageal squamous cell carcinoma (Komatsu et al., 2012), prostate cancer (Yaman Agaoglu et al., 2011), and glioblastoma (Skog et al., 2008).

Skog et al. (2008) previously reported that glioblastoma tumor cells release EVs containing mRNA, miRNA, and angiogenic proteins, and these EVs are taken up by normal host cells, such as brain microvascular endothelial cells. These authors also showed that miR-21 levels are elevated in serum EVs from glioblastoma patients compared with controls. Circulating miR-21 has been reported in the serum/plasma obtained from various cancer patients, although the contribution of miRNAs to cancer development through EVs has not been discerned. miR-21 acts as an oncogenic miRNA in various cancer cells and also regulates various phenotypes in the cancer cell microenvironment. Indeed, miR-21 is not only involved in cancer development but also participates in homeostasis (Niu et al., 2011); thus, understanding the contribution of miR-21 to the cellular microenvironment will increase the global understanding of animal development.

miR-21, associated with RNA-binding proteins, has also been detected in the culture supernatant from breast cancer cell lines (Turchinovich et al., 2011) and serum from healthy donors (Arroyo et al., 2011), and the abundance of miR-21 in the extracellular space has been recognized as shown above. Thus, determining the biological significance for miR-21 binding to Ago2 might provide a better understanding of miRNA-associated cell–cell communication in cancer development.

## miR-126

One of the earliest studies to show the transfer of miRNAs between the cells was revealed by the study of apoptotic bodies. In this study, the authors found that endothelial cell-derived apoptotic bodies contained miR-126 and these apoptotic bodies convey paracrine alarm signals to recipient vascular cells during atherosclerosis (Zernecke et al., 2009). In addition, another study also showed that secretory miR-126 was precipitated in the angiogenesis. The EVs from CD34^+^ peripheral blood mononuclear cells exhibited proangiogenic properties via the transfer of miR-126 (Mocharla et al., 2013). Cantaluppi et al. (2012a) reported that EVs released from endothelial progenitor cells (EPCs) enhanced islet endothelial cell proliferation, migration, anti-apoptosis, and organization in vessel-like structures. They also found that EVs from EPCs contained the miR-126 and miR-296 and that these miRNAs contributed to the angiogenesis properties, suggesting that EVs from EPCs activate an angiogenic program in islet endothelium (Cantaluppi et al., 2012a). They also reported that miR-126 in EVs from EPCs contributed to the prevention of the ischemic acute injury in kidney by enhanced tubular cell proliferation, reduced apoptosis, and leukocyte infiltration (Cantaluppi et al., 2012b). In addition, EPC-derived EVs were able to induce neoangiogenesis and to enhance recovery in a hindlimb ischemia (Ranghino et al., 2012).

Although circulating miR-126 was enriched in systemic lupus erythematosus (Wang et al., 2012a), expression of circulating miR-126 was decreased in the breast cancer (Wang et al., 2010) and malignant mesothelioma (Tomasetti et al., 2012). Whereas there are only a few reports regarding the circulating miR-126, secretory miR-126 from cells has a great activity of endothelial cells activations as shown in above. Therefore, it is tempting to investigate the potential of miR-126 as biomarker in diseases which were caused by the abnormal angiogenesis.

## miR-451

Kogure et al. (2011) showed a subset highly enriched miRNAs within EVs from HCC cells and identified a target of these miRNAs, transforming growth factor β activated kinase-1. Indeed, loss of this pathway resulted in the enhancement of transformed cell growth in recipient cells. One of the miRNAs that they identified in this study, miR-451, was found in the serum from patient with liver disease. Murakami et al. (2012) investigated the disease parameters in patients with chronic hepatitis C (CHC) by focusing on miRNAs isolated from EV-enriched fraction in serum. They successfully classified CHC and normal liver with 96.59% accuracy using the expression patterns of nine miRNAs including miR-451 (Murakami et al., 2012).

## miR-223

Ismail et al. (2013) found that EVs from macrophage contained miR-223, and that this miR-223 was transported to target cells, including monocytes, endothelial cells, epithelial cells, and fibroblasts, and was functionally active. Macrophages are found in all tissues and they play roles in development, homeostasis, tissue repair, and immunity, and thus are therapeutic targets in many human diseases (Wynn et al., 2013). Indeed, an increased level of circulating miR-223 was found in serum/plasma from patients with gastric cancer (Li et al., 2012), non-small cell lung carcinoma (Sanfiorenzo et al., 2013), hepatitis B virus-related HCC (Zhou et al., 2011), NPC (Zeng et al., 2012), hypertension-induced heart failure (Dickinson et al., 2013), systemic lupus erythematosus, rheumatoid arthritis (Wang et al., 2012a), sepsis (Wang et al., 2012b), ischemic injury (Yu et al., 2009), and osteoarthritis (Okuhara et al., 2012). To date, origins of this circulating miR-223 have not been investigated yet; however, from the reports shown above, macrophage is probable candidate of origin for circulating miR-223. Interestingly, miR-223 is found not only in EVs but also in HDL (Vickers et al., 2011). In addition, miR-223 concentration in HDL was increased 3,780-fold with familial hypercholesterolemia when compared with controls. The HDL is involved in the transport of cholesterol from lipid-enriched macrophages of atherosclerotic arteries to the liver. Recently, Wagner et al. (2013) reported that miR-223 was detected at concentrations >10,000 copies/μg in HDL from healthy subjects. However, HDL-bound miR-223 contributed to only 8% of the total circulating miRNAs. In addition, a significant uptake of HDL-bound miRNAs into endothelial cells, smooth muscle cells, or peripheral blood mononuclear cells was not observed, suggesting that the lipoprotein-associated miR-223 does not regulate the function of the studied cells *in vitro*. Knowing the function of secretory miR-223 in macrophage homeostasis *in vivo* might lead to the development of not only the disease biomarker, but also the novel therapy against atherosclerosis.

## miR-150

Zhang et al. (2010) demonstrated that miR-150 from monocytic cells were delivered into endothelial cell, and this miR-150 reduced its target gene, c-Myb, expression in endothelial cells, resulting in the enhancement of cell migration in endothelial cell both *in vitro* and *in vivo*. They also found that monocyte-secreted miR-150 promoted angiogenesis *in vivo* using tumor-implanted mice and ob/ob mice as models (Li et al., 2013). Intriguingly, the expression of miR-150 was higher in EVs isolated from the plasma of patients with atherosclerosis, and these EVs promoted endothelial cell migration compared to EVs from healthy donors (Zhang et al., 2010). A high level of circulating miR-150 was reported in several diseases including idiopathic childhood nephrotic syndrome (Luo et al., 2013), acute myeloid leukemia (Fayyad-Kazan et al., 2013), and so on. On the contrary, miR-150 serum concentrations upon admission were closely associated with intensive care unit (ICU) survival as well as long-term survival, and low miR-150 levels indicated an unfavorable prognosis (Roderburg et al., 2013).

## SUMMARY AND PERSPECTIVES

In this review, we presented the results obtained from research on miRNAs to provide a better understanding of the relationship between secreted miRNAs which contribute to cell–cell communication in cancer development, and circulating miRNAs which are used as disease biomarkers.

Recently, a novel concept for biomarkers, called “liquid biopsy,” has been proposed (Forshew et al., 2012; Murtaza et al., 2013). Liquid biopsy would be useful for numerous diagnostic applications and avoid the need for tumor tissue biopsies. Current studies have shown that genomic alterations in solid cancer can be characterized through the massively parallel sequencing of circulating cell-free tumor DNA released from cancer cells into the plasma (Forshew et al., 2012; Murtaza et al., 2013). This suggests that circulating miRNAs are also good candidates for liquid biopsy, as the quantities and sequences of miRNAs convey information for diagnosis. Particularly, circulating miRNAs, which have been previously shown to function in cell–cell communication, might be good candidates for this application. Therefore, we emphasize that it is important to investigate the function of secretory miRNAs in cell–cell communication, and in parallel explore the usefulness of these molecules as biomarkers using animal models.

Much of the current research on circulating miRNAs for disease biomarkers does not describe the types of circulating miRNAs, such as EVs, microvesicles, HDL/LDLs, or RNA-binding proteins that are present in human body fluids. As previously discussed, focusing on a specific type of circulating miRNAs, such as exosomal miRNAs or miRNAs bound to RNA-binding proteins, might be useful as disease biomarkers compared with analyzing the total miRNA in human body fluids. Indeed, EV-enriched fractions isolated from patients with liver disease were useful for the determination of disease progression compared with the profiles obtained using total miRNA present in serum samples (Murakami et al., 2012). Therefore, it is essential that future studies concerning circulating miRNAs for diagnostic purposes should focus on the type of circulating miRNAs present in body fluids.

One of the crucial issues in research on cell–cell communication by secretory miRNAs is whether the secretory miRNAs which researcher identified are really physiologically functional enough or not. This issue might be revealed by showing the quantitative data of secretory miRNAs in more detail, such as the number of EVs, the number of miRNAs, and the number of cells. In addition, in the case of functional demonstration of secretory miRNAs, over-expression or knock-down of secretory miRNAs was performed; however, contamination of exogenous miRNAs, such as synthetic miRNAs, should be cared since the amount of those exogenous miRNAs are usually introduced in excess. The study on extracellular miRNAs has just begun. Thus, the researcher working on the EVs needs to take care of the physiological amount of those molecules in their research field.

Another crucial issue of extracellular miRNAs that how these miRNAs are secreted from cells and how these miRNAs work in the cells has not been answered yet, although recent reports proved the physiological and pathological importance of secretory miRNAs not only *in vitro* but also *in vivo*. We previously found that secretion of miRNAs from cells was regulated by neutral sphingomyelinase 2, which is known as a rate-limiting enzyme of ceramide biosynthesis and triggers secretion of EVs (Kosaka et al., 2010b). Although the molecules that are essential for EVs secretion has been reported, their contribution to miRNAs secretion has not been tested yet. One of the most important points for understanding of miRNAs secretion is the identification of a protein that binds to miRNAs in EVs. miRNAs are strongly bound to the Ago2 protein, which is a main component of the RNA-induced silencing complex (RISC), in the cells (Kim et al., 2009), but this molecule is not found in EVs (Gibbings et al., 2009). Meanwhile, knockdown of GW182, another main component of the RISC, reduced miRNA secretion via EVs. Interestingly, however, GW182 was not detected in the EVs from HEK293 (Yao et al., 2012). In contrast to the above report, GW182 can be found in EVs from monocyte, HeLa cells and *ex vivo*-derived dendritic cells (Gibbings et al., 2009). These paradoxical observations indicate that further experiments are required to elucidate whether there is a role for GW182 in miRNA secretion. Identification of proteins that are responsible for the transport of miRNAs from inner cells to inner EVs might reveal many of mysteries of secretory miRNAs in cell–cell communications.

Circulating RNA has been previously considered as “trash” from cells; however, we propose that this “trash” serves as a communication tool and should therefore be referred to as “treasure.” Analyzing circulating miRNAs in human body fluids might provide a method for “listening” to the communication between cells, leading to the development of disease treatments based on the mechanisms of secreted miRNAs in cancer development.

## Conflict of Interest Statement

The authors declare that the research was conducted in the absence of any commercial or financial relationships that could be construed as a potential conflict of interest.
